# Risk Factors of Clinically Relevant Upper Gastrointestinal Bleeding in Cardiovascular Intensive Care Unit

**DOI:** 10.21470/1678-9741-2025-0260

**Published:** 2026-09-08

**Authors:** Aytac Polat, Selcuk Gulmez

**Affiliations:** 1 Department of Anesthesia and Reanimation, University of Health Sciences, Kosuyolu Higher Specialty Training and Research Hospital, Istanbul, Turkey.; 2 Department of Gastrointestinal Surgery, University of Health Sciences, Kosuyolu Higher Specialty Training and Research Hospital, Istanbul, Turkey.

**Keywords:** Upper Gastrointestinal Bleeding, Critical Illness, Anticoagulant, Antiplatelet, Antithrombotic Therapy

## Abstract

**Introduction:**

Upper gastrointestinal bleeding (UGIB) is a serious but underexplored complication in cardiovascular intensive care unit (CV-ICU) patients, with often self-limiting, yet clinically relevant, worsening outcomes.

**Objective:**

This study aimed to identify independent predictors of new-onset UGIB in CV-ICU patients.

**Methods:**

In this retrospective cohort study, 50 patients who underwent bedside upper gastrointestinal endoscopy in the CV-ICU due to overt UGIB were analyzed. Clinical data, laboratory parameters, and intensive care unit (ICU)-related variables were compared between patients with clinically relevant and non-relevant UGIB. Logistic regression analyses were performed to identify independent risk factors.

**Results:**

Clinically significant UGIB occurred in nine patients (18%), with a mortality rate of 77.8% (7/9). Multivariate analysis revealed that Acute Physiology and Chronic Health Evaluation (APACHE) II score (odds ratio [OR] 1.47, *P* = 0.018), Sequential Organ Failure Assessment (SOFA) score (OR 1.08, *P* = 0.045), and ICU stay before bleeding (OR 1.49, *P* = 0.049) were independent risk factors. Receiver operating characteristic analysis showed strong discriminative ability of APACHE II (area under the curve [AUC] 0.927, sensitivity 100%, specificity 85.4%), whereas SOFA demonstrated moderate performance (AUC 0.772, sensitivity 77.8%, specificity 78.0%). The use of anticoagulants, antiplatelets, or dual therapy was not significantly associated with clinically relevant bleeding (*P* > 0.05).

**Conclusion:**

Higher SOFA and APACHE II scores, as well as prolonged ICU stay, were independent risk factors for clinically significant new-onset UGIB in CV-ICU patients. APACHE II provided superior predictive accuracy compared with SOFA. Clinically relevant UGIB was strongly linked to mortality, while antithrombotic therapy type showed no significant effect.

## INTRODUCTION

Patients admitted to the cardiovascular intensive care unit (CV-ICU) represent a highly vulnerable cohort due to complex comorbidities and elevated risk of perioperative complications. Management in the CV-ICU focuses on cardiac and vascular conditions, particularly in the context of surgical interventions, including aortic, valvular, coronary, and peripheral vascular surgeries. The widespread use of antithrombotic agents in this population further compounds their susceptibility to bleeding events.

Upper gastrointestinal bleeding (UGIB) is a serious intensive care unit (ICU) complication, presenting as hematemesis or melena and occasionally causing acute anemia. Bedside esophagogastro-duodenoscopy (EGD) aids in diagnosis and treatment^[1]^. Though clinically significant UGIB is uncommon, it can worsen outcomes. Some studies associate UGIB with high mortality^[2]^, while others find no link to 90-day mortality^[3]^. UGIB often resolves without major deterioration. However, predicting its clinical relevance remains challenging^[4]^.

Well-established risk factors for UGIB include physiological stress, cardiovascular disease, critical illness, and ICU interventions, especially when combined with anticoagulants or antiplatelets^[5]^. Additional pathophysiological mechanisms in the CV-ICU setting involve splanchnic hypoperfusion due to hemodynamic instability and vasopressor use, stress-related mucosal damage aggravated by mechanical ventilation (MV), and the effect of routine anti-thrombotic therapy, which further increases bleeding risk once mucosal injury occurs. The role of MV remains controversial, with conflicting evidence in the literature^[6,7,8]^. Similarly, prolonged ICU stays have been proposed as a risk factor^[9]^, though evidence is inconsistent^[10]^. Overall, the literature on clinically significant UGIB remains limited.

In CV-ICU patients, UGIB may adversely affect clinical outcomes. Identifying risk factors is essential to guide preventive strategies, optimize management, and improve prognosis. Accordingly, this study aimed to investigate predictors of clinically relevant UGIB in CV-ICU patients.

## METHODS

### Ethics Approval

The study was approved by the Institutional Review and Ethics Committee of University of Health Sciences, Kosuyolu Higher Specialty Training and Research Hospital, Istanbul, Turkey (approval No: 2025/01/1026) and conducted in accordance with the Declaration of Helsinki. Written informed consent was obtained from all patients for surgery and use of clinical data.

### Patient Selection and Data Source

This retrospective cohort study was conducted at the CV-ICU of Kosuyolu Higher Specialty Training and Research Hospital, a tertiary referral center in Istanbul, Turkey. Among 2,760 patients admitted between January 1, 2020, and October 31, 2024, 50 who developed UGIB were included (prevalence 1.8%). The study employed a total population (convenience) sampling approach, including all eligible patients who met the predefined inclusion and exclusion criteria based on medical record review.

All consecutive eligible patients during this period were analyzed. Inclusion criteria were patients aged ≥ 18 years who underwent cardiovascular surgery, deteriorated preoperatively, and were admitted to CV-ICU, or were monitored in CV-ICU after cardiovascular interventions. Exclusion criteria were non-cardiovascular surgery, patients not receiving any antithrombotic therapy during their CV-ICU stay, EGD performed outside the CV-ICU, or the presence of lower gastrointestinal bleeding (see Figure 1 for patient selection flowchart).

**Fig. 1:**
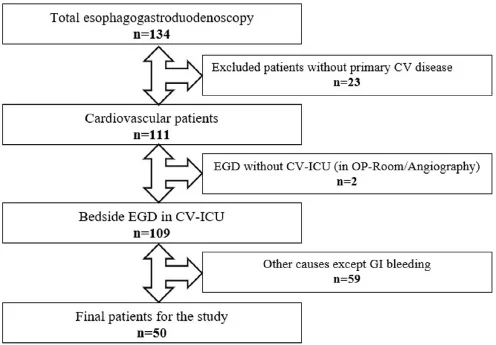
Demonstration of patients undergoing bedside esophagogastroduodenoscopy (EGD) in the cardiovascular intensive care unit (CV-ICU). CV=cardiovascular; GI=gastrointestinal; OP=operation.

### Study Parameters

Collected data included age, sex, ejection fraction (EF), interventions, 30-day mortality, antithrombotic use, EGD indication, and endoscopic findings. Clinically relevant *vs.* non-relevant UGIB cases were compared by age, sex, the International Normalized Ratio (INR), platelets, EF, Acute Physiology and Chronic Health Evaluation (APACHE) II score, Sequential Organ Failure Assessment (SOFA) score, use of steroids or vasopressors, MV, interventions, length of stay (LOS) in ICU, 30-day mortality, and surgery status.

### Definitions

The non-operative group comprised preoperative deteriorations, failed interventions referred to surgery, and certain procedures like thoracic endovascular aortic repair. Readmissions within 30 days for reasons other than gastrointestinal bleeding were also included. MV duration refers to uninterrupted ventilation before EGD. LOS represents the time from ICU admission to EGD. UGIB was defined as new-onset bleeding originating from the esophagus, stomach, or duodenum, proximal to the ligament of Treitz^[11]^. Overt UGIB included hematemesis, melena, or bloody nasogastric aspirate. Clinically significant UGIB was defined as overt bleeding with a hemoglobin drop of ≥ 2 g/dL or a hematocrit drop of ≥ 6%, together with hemodynamic instability^[12]^. All EGDs were performed within 24 hours of bleeding onset. All patients received intravenous proton pump inhibitor (PPI) (pantoprazole, 40 mg once daily) upon ICU admission.

### Statistical Analysis

Statistical analyses were performed using IBM SPSS Statistics for Windows, version 22 (IBM Corp., Armonk, N.Y., USA). Qualitative variables were expressed as frequencies and percentages. The distribution of numerical variables was tested using the Shapiro– Wilk test. Since several continuous variables were not normally distributed, they were presented as median with interquartile range (IQR) and compared using the non-parametric Mann–Whitney U test. Categorical variables were analyzed using chi-square or Fisher’s exact test. Risk factors were assessed with univariate and multivariate logistic regression. A *P*-value < 0.05 was considered statistically significant.

## RESULTS

Demographic and clinical data for all patients are presented in Table 1. The median age (IQR) of these patients was 65 (53 - 69) years. The majority of patients were male (n = 40, 80%). The non-operative group comprised 18 patients (36%). In comparison, 13 patients (26%) underwent valvular surgery, 10 patients (20%) underwent coronary artery bypass grafting surgery, four patients (8%) had aortic surgery, four patients (8%) received left ventricular assist device, and one patient (2%) was diagnosed with peripheral arterial disease. Overall mortality was 30% (15 patients).

**Table 1.** Demographic and clinical data of all patients (n = 50).

		Median (IQR)
Age, years		65 (53 - 69)
		**n (%)**
Sex	Male	40 (80)
	Female	10 (20)
Interventions	Non-operative CV patients	18 (36)
	Valvular surgery	13 (26)
	CABG	10 (20)
	Aortic surgery	4 (8)
	LVAD	4 (8)
	PAD	1 (2)
30-day mortality	No	35 (70)
	Yes	15 (30)

CABG=coronary artery bypass grafting; CV=cardiovascular; IQR=interquartile range; LVAD=left ventricular assist device; PAD=peripheral arterial disease

Table 2 outlines the antithrombotic medications used by patients who underwent bedside endoscopy in the CV-ICU. Half of the patients were treated with single acetylsalicylic acid therapy. Of the total, 38 patients (76%) were on single-agent treatment, while 12 (24%) received at least dual-agent therapy. Additionally, 17 (34%) patients were on single or combined warfarin therapy, 30 (60%) patients were on antiplatelet therapy, nine (18%) patients were on anticoagulant therapy, and 11 (22%) patients were receiving combination therapy.

**Table 2.** Antithrombotic agents of all patients (n = 50).

	n (%)
ASA	25 (50)
Warfarin	8 (16)
ASA + warfarin	7 (14)
Clopidogrel	4 (8)
ASA + bivalirudin	2 (4)
ASA + clopidogrel + warfarin	2 (4)
ASA + clopidogrel	1 (2)
Bivalirudin	1 (2)

ASA=acetylsalicylic acid

Table 3 presents the indications and findings of endoscopy. The primary indication for bedside EGD was melena (66%), followed by hematemesis (28%), and blood detected in a nasogastric tube (6%). Regarding endoscopic findings, gastric or duodenal erosions and antral gastritis accounted for 74% of the cases.

**Table 3.** Indications and findings in endoscopy of patients with new-onset UGIB (n = 50).

Indications	n (%)	Findings	n (%)
Melena	33 (66)	Gastric or duodenal erosions	20 (40)
Hematemesis	14 (28)	Antral gastritis	17 (34)
Blood in nasogastric tube	3 (6)	Peptic ulcer disease	6 (12)
		Pangastritis	5 (10)
		Aortoenteric fstula	1(2)
		Esophagitis	1 (2)

UGIB=upper gastrointestinal bleeding

Clinically relevant UGIB occurred in nine patients (18%) (Table 4). There were no significant differences between relevant and non-relevant UGIB in age, sex, EF, INR, platelet count, type of surgery, or corticosteroid use. Antithrombotic regimen type (single *vs.* dual) and specific agents (*e.g.*, warfarin) did not correlate with clinically relevant UGIB. However, MV use (*P* = 0.028), vasopressor therapy (*P* = 0.015), higher APACHE II scores (22 *vs.* 9, *P* < 0.001), SOFA scores (12 *vs.* 5, *P* = 0.010), and longer ICU LOS (47 *vs.* 9 days, *P* = 0.032) were significantly associated with clinically relevant UGIB. Seven of the 15 patients (46.7%) who died had clinically relevant UGIB (*P* = 0.002).

**Table 4.** Data of patients with clinically relevant UGIB.

		Clinically relevant UGIB		
		No (n = 41, 82%)	Yes (n = 9, 18%)	
		Median (IQR) or n (%)	Median (IQR) or n (%)	*P*-value
Age, years		65 (53 - 68)	63 (62 - 71)	0.426
Sex	Male	32 (80)	8 (20)	0.661
	Female	9 (90)	1 (10)	
INR		1.3 (1.1 - 1.6)	1.2 (1.1 - 1.5)	0.960
Platelet, ×10³/µL		197 (118 - 250)	87 (61 - 229)	0.193
EF, %		65 (55 - 65)	65 (50 - 65)	0.766
APACHE II score		9 (7 - 16)	22 (20 - 24)	< 0.001
SOFA score		5 (2 - 8)	12 (9 - 17)	0.010
Corticosteroid	No	38 (84.4)	7 (15.6)	0.216
	Yes	3 (60)	2 (40)	
Vasopressors	No	32 (91.4)	3 (8.6)	**0.015**
	Yes	9 (60)	6 (40)	
Mechanical ventilation	No	22 (95.7)	1 (4.3)	**0.028**
	Yes	19 (70.4)	8 (29.6)	
≥ 2 antithrombotic agents	No	32 (84.2)	6 (15.8)	0.668
	Yes	9 (75)	3 (25)	
Warfarin single/ combined	No	28 (84.8)	5 (15.2)	0.467
	Yes	13 (76.5)	4 (23.5)	
Antithrombotic	Anticoagulant	7 (77.8)	2 (22.2)	0.551
	Antiplatelet	26 (86.7)	4 (13.3)	
	Combined	8 (72.7)	3 (27.3)	
Surgery	No	16 (88.9)	2 (11.1)	0.459
	Yes	25 (78.1)	7 (21.9)	
Interventions	Non-operative	16 (88.9)	2 (11.1)	0.359
	Valvular surgery	11 (84.6)	2 (15.4)	
	CABG	8 (80)	2 (20)	
	Aortic surgery	3 (75)	1 (25)	
	LVAD	3 (75)	1 (25)	
	PAD	0 (0)	1 (100)	
LOS, days		9 (5 - 14)	47 (11 - 57)	**0.032**
30-day mortality	No	33 (94.3)	2 (5.7)	**0.002**
	Yes	8 (53.3)	7 (46.7)	

APACHE=Acute Physiology and Chronic Health Evaluation; CABG=coronary artery bypass grafting; EF=ejection fraction; INR=Inter-national Normalized Ratio; IQR=interquartile range; LOS=length of stay; LVAD=left ventricular assist device; PAD=peripheral arterial disease; SOFA=Sequential Organ Failure Assessment; UGIB=upper gastrointestinal bleeding

Receiver operating characteristic (ROC) analysis (Figure 2) demonstrated that both APACHE II and SOFA scores were predictive of clinically relevant UGIB. APACHE II demonstrated excellent discrimination (area under the curve [AUC]: 0.927, 95% confidence interval [CI]: 0.854 – 1.000, *P* < 0.001) with 100% sensitivity and 85.4% spec-ificity at a cutof of 19.5. In contrast, SOFA showed only acceptable performance (AUC: 0.772, 95% CI: 0.564 – 0.981, *P* = 0.011). Overall, APACHE II proved superior for risk stratification in CV-ICU patients. In multivariate logistic regression (Table 5), APACHE II (odds ratio [OR]: 1.47, *P* = 0.018), SOFA score (OR: 1.08, *P* = 0.045), and LOS (OR: 1.49, *P* = 0.049) emerged as independent predictors. MV and vasopressors showed significance in univariate but not in multi-variate analysis.

**Fig. 2:**
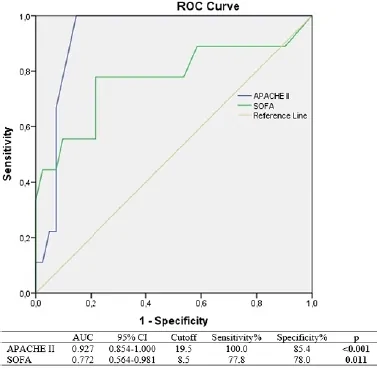
Receiver operating characteristic (ROC) analysis of Acute Physiology and Chronic Health Evaluation (APACHE) II and Sequential Organ Failure Assessment (SOFA) scores for clinically relevant upper gastrointestinal bleeding. AUC=area under the curve; CI=confidence interval.

**Table 5.** The risk factors for clinically relevant UGIB by univariate and multivariate regression analyses.

	Clinically relevant UGIB			
	Univariate		Multivariate	
	OR (95% CI)	*P*-value	OR (95% CI)	*P*-value
APACHE II score	1.345 (1.108 - 1.634)	**0.003**	1.470 (1.067 - 2.025)	**0.018**
LOS, days	1.077 (1.025 - 1.131)	**0.003**	1.490 (1.001 - 2.217)	**0.049**
SOFA score	1.281 (1.081 - 1.519)	**0.004**	1.084 (1.002 - 1.173)	**0.045**
Vasopressor, yes	7.111 (1.478 - 34.210)	**0.014**	0.224 (0.003 - 17.515)	0.501
Mechanical ventilation, yes	9.263 (1.060 - 80.934)	**0.044**	0.146 (0.005 - 4.050)	0.256

APACHE=Acute Physiology and Chronic Health Evaluation; CI=confidence interval; LOS=length of stay; OR=odds ratio; SOFA=Sequential Organ Failure Assessment; UGIB=upper gastrointestinal bleeding

## DISCUSSION

This study showed that SOFA score, APACHE II score, and LOS in CV-ICU are independent risk factors for clinically significant new-onset UGIB. Additionally, these patients had higher mortality. However, no significant association was found between anti-thrombotic therapy type (anticoagulant *vs.* antiplatelet) or single *vs.* dual therapy and clinically relevant UGIB.

UGIB is a frequent concern in ICUs, though most cases resolve without major complications. Up to 70% of UGIBs, even with dual antiplatelet therapy, may not be clinically relevant^[13]^. Nevertheless, severe or life-threatening bleeding can occur in critically ill patients. One study found clinically significant UGIB in 3.5% of ICU patients^[14]^, while rates may reach 16% with antithrombotic use^[15]^. In our study, nine of 50 patients (18%) undergoing bedside EGD had clinically significant bleeding; seven had prior surgery. However, surgery was not statistically associated with significant UGIB. UGIB-related mortality varies. A recent study reported 11.4% UGIB incidence with 63.4% mortality in bleeding cases^[10]^, while Kim et al.^[16]^ observed 44.7% in-hospital mortality in ICU patients with UGIB. A previous study found a 90% mortality rate in clinically significant gastrointestinal bleeding (both upper and lower) despite PPI use^[17]^. Conversely, Rao et al.^[18]^ found that among 188 ICU patients with new-onset UGIB, only three of 32 deaths were attributable to bleeding. In our cohort, seven of nine patients (77.8%) with clinically relevant UGIB died. Similar to Sakai et al.^[19]^, we found no association between the type or number of antithrombotic agents and bleeding. Although a current study suggests a higher bleeding risk with combined therapy^[20]^, our subgroup analysis did not confirm this.

ROC analysis demonstrated that the SOFA score had acceptable discriminative ability (AUC: 0.772), indicating that although useful for risk stratification, its predictive performance was inferior to that of the APACHE II score. In logistic regression analysis, the SOFA score remained significantly associated with clinically relevant UGIB (OR: 1.08, 95% CI: 1.002 – 1.173, *P* = 0.045), consistent with Granholm et al.^[21]^ findings (OR 1.13 per point; OR 1.88 per fve points). Sakai et al.^[19]^ also noted higher SOFA scores in bleeding patients (4.3 ± 3.6 *vs.* 2.8 ± 3.2, *P* = 0.016), particularly in critically ill cardiovascular cases. Higher SOFA scores indicate the extent of organ dysfunction and the accompanying inflammatory response, predisposing patients to erosive gastropathy, ulcer formation, and UGIB^[22,23]^.

APACHE II score was another independent predictor (OR: 1.47, *P* = 0.018). Median scores were significantly higher in the relevant UGIB group (22 *vs.* 9, *P* < 0.001). Furthermore, ROC analysis con-firmed the superior discriminative capacity of APACHE II (AUC: 0.927), with excellent sensitivity and specificity, supporting its role as the most reliable predictor of clinically relevant UGIB in CV-ICU patients. While older studies found no difference^[14]^, more recent data identified APACHE II > 14 as a significant risk factor^[10]^. A high APACHE II score reflects dysfunction in multiple organ systems, potentially indicating hypovolemia, hypoxia, and systemic inflam-mation, all of which can lead to impaired gastric mucosal barrier function and increase the risk of gastrointestinal hemorrhage.

Longer ICU stay before UGIB was the third independent risk factor (OR: 1.49, 95% CI: 1.001 – 2.217, *P* = 0.049). Median LOS was significantly higher in the clinically relevant group (47 *vs.* 9 days, *P* = 0.032), consistent with earlier findings^[10,24]^. Kumar et al.^[9]^ reported ICU stays > 11 days as a strong predictor (OR: 14.7). Extended stays increase exposure to risk factors like MV, vasopressors, sepsis, and coagulopathy^[25]^.

Our findings highlight preventive strategies for clinically relevant UGIB in CV-ICU patients. Emphasis should be placed on early management of organ dysfunction and the prompt identification of modifiable risk factors. Practical measures include optimizing hemodynamics, minimizing unnecessary MV, cautious vasopres-sor use, and reducing ICU stay. In addition, timely stress ulcer prophylaxis and close monitoring of high-risk patients are crucial to mitigating UGIB risk.

This study uniquely focused on clinically relevant UGIB in a cardiovascular ICU population. It analyzed antithrombotic subtypes, surgical status, and LOS as a predictor, unlike many studies that report LOS as a consequence.

### Limitations

Limitations include the single-center, retrospective design, and the relatively small sample size. Because all patients received antithrombotics, direct comparison with untreated patients was not possible. However, the subgroup distribution of antithrombotic therapy was reported. In addition, intraoperative factors were not fully analyzed. Future studies should investigate surgical influences and the impact of modifying or temporarily withholding antithrombotics to better guide clinical practice.

## CONCLUSION

In this retrospective cohort of CV-ICU patients, clinically relevant UGIB occurred in nearly one-fifth of cases and was strongly associated with mortality. MV and vasopressor use were significant in univariate analysis. However, in multivariate regression, disease severity (APACHE II and SOFA scores) and longer ICU stay emerged as independent predictors. ROC analysis confirmed these results, with APACHE II demonstrating superior discriminative accuracy compared with SOFA. Overall, clinically relevant UGIB appears to be driven primarily by critical illness severity rather than anti-thrombotic therapy. Early recognition and targeted management of high-risk patients may help improve outcomes.

## Data Availability

The authors declare that they did not deposit the study data in a public repository. Because the dataset contains retrospective clinical data obtained from patient medical records, public sharing is restricted to protect patient confdentiality and comply with institutional regulations. The datasets generated and/or analyzed during the current study are available from the corresponding author upon reasonable request.
